# Simple Determination of Affinity Constants of Antibodies by Competitive Immunoassays

**DOI:** 10.3390/mps7030049

**Published:** 2024-06-13

**Authors:** Janina Fischer, Jan Ole Kaufmann, Michael G. Weller

**Affiliations:** 1 Federal Institute for Materials Research and Testing (BAM), Richard-Willstätter-Strasse 11, 12489 Berlin, Germany; 2 Department of Chemistry, Humboldt-Universität zu Berlin, Brook-Taylor-Straße 2, 12489 Berlin, Germany; 3 Charité—Universitätsmedizin Berlin, Charitéplatz 1, 10117 Berlin, Germany

**Keywords:** ELISA, competitive immunoassay, IC_50_, test midpoint, point of inflection, equilibrium constant, dissociation constant, intrinsic affinity, surface–plasmon resonance, SPR, interaction, K_d_ value

## Abstract

The affinity constant, also known as the equilibrium constant, binding constant, equilibrium association constant, or the reciprocal value, the equilibrium dissociation constant (K_d_), can be considered as one of the most important characteristics for any antibody–antigen pair. Many methods based on different technologies have been proposed and used to determine this value. However, since a very large number of publications and commercial datasheets do not include this information, significant obstacles in performing such measurements seem to exist. In other cases where such data are reported, the results have often proved to be unreliable. This situation may indicate that most of the technologies available today require a high level of expertise and effort that does not seem to be available in many laboratories. In this paper, we present a simple approach based on standard immunoassay technology that is easy and quick to perform. It relies on the effect that the molar IC_50_ approaches the K_d_ value in the case of infinitely small concentrations of the reagent concentrations. A two-dimensional dilution of the reagents leads to an asymptotic convergence to K_d_. The approach has some similarity to the well-known checkerboard titration used for the optimization of immunoassays. A well-known antibody against the FLAG peptide, clone M2, was used as a model system and the results were compared with other methods. This approach could be used in any case where a competitive assay is available or can be developed. The determination of an affinity constant should belong to the crucial parameters in any quality control of antibody-related products and assays and should be mandatory in papers using immunochemical protocols.

## 1. Introduction

Affinity constants are one of the most crucial characteristics of antibodies, since many performance parameters of immunoassays and even in therapeutics are strongly linked to this value [1,2]. Furthermore, in the context of the reproducibility crisis, antibody characterization came into focus [3]. Affinity constants are often listed as the most crucial parameters for antibody validation [4] and hence, experimental protocols [5] seem to be incomplete, as long as affinity constants are not provided. Surprisingly, affinity constants are nevertheless neglected in many papers. Even when an affinity constant is given, the value is often unreliable or ambiguous. Many researchers are not aware of the relevance and complexity of affinity determinations and of the vast number of pitfalls in this context. It is not possible to discuss all these issues in this paper. Here, we want to focus on a practical solution which makes it easier for the researcher to determine useful affinity constants without excessive efforts. In addition, this paper cannot give a comprehensive review of all methods based on commercial or academic devices. The reader is referred to excellent reviews [6,7,8] which have been published previously. It also has to be noted that we do not claim to determine a “true” thermodynamic equilibrium constant, which is very difficult to measure in the strict sense [9]. The determination of “true” binding constants would often require research projects on their own. To assume that these efforts can be spent in a typical biomedical and biochemical lab seems to be unrealistic. However, we think that the deviation from our values to this “true” constant is not critical in most cases, particularly if the broad range of values is considered, which is obtained by different methods and replicated measurements.

Today, only a few methods for affinity determinations seem to have some practical relevance. In a limited field, the application of isothermal titration microcalorimetry (ITC) is considered the gold standard. A significant advantage is its universality and the homogeneous and label-free reaction [10,11]. This avoids many of the problems encountered in heterogeneous systems. Additional thermodynamic data can be extracted from these measurements. Unfortunately, this approach is not very sensitive and hence consumes large amounts of reagents, which is prohibitive for many applications. Therefore, it seems to be not very popular for the characterization of expensive antibodies and their antigens.

During the last decades, surface plasmon resonance (SPR) has developed into the most dominant method for the determination of affinity constants [12,13]. This method is also known as the Biacore^TM^ method, based on the former name of a leading company offering such systems. As the term suggests, SPR is a surface method using a gold layer with attached reagents. Many different types of pre-coated SPR chips are commercially available, which makes their application quite accessible. However, most users greatly underestimate the pitfalls associated with this technology. The main drawback is the heterogenous assay, which may lead to severe dissociation limitations, particularly if thicker layers (“3D”) are used to achieve higher signals. The main advantage of the method is the fact that it can be considered label-free, and hence it is assumed that the undisturbed affinity constant could be obtained in contrast to other methods, which rely on labeled reagents. However, from our point of view, this is only half of the story. In all cases, one of the reagents needs to be immobilized on the SPR chip, which is often even more disturbing as the attachment of a radioisotope, dye, or enzyme. With SPR, not only thermodynamic equilibrium constants can be determined, but also kinetic binding constants, such as the on-rate and off-rate constant. This is definitely a big plus of SPR. Unfortunately, these data can also be distorted by unwanted surface or diffusion effects. In addition, label-free methods are severely hampered by any non-specific binding of irrelevant materials and, in the case of SPR, by even minimal changes in the refractive index. Reference channels can only partially compensate for these interferences. Finally, if antigen-coated chips are used, often multivalence effects [14] may lead to tremendous overestimations of the monovalent binding strength of an antibody to its antigen. Some authors refer to monovalent affinity as “intrinsic affinity” [15,16]. In contrast, the functional affinity, including multivalence and other complex mechanisms, is known as avidity. This number may have some relevance in special situations, but it should not be mixed up with the traditional affinity constant because this can lead to severe misinterpretations, as seen in many papers. Nevertheless, SPR is the most frequently used method for the determination of affinity constants today, irrespective of the repeated critical discussions [17,18,19]. Also, a similar technique, known as bio-layer interferometry (BLI) [20], is commercialized in Octet systems. Other devices based on SPR have been discussed in a recent review [21]. In silico prediction models for affinity constants are in development [22,23], although their precision seems to be not sufficient for routine applications, yet.

As mentioned above, multivalency, in the case of IgG the bivalency of antibodies, is a severe issue [24]. The multivalency of antibodies was discussed in detail by Crothers and Metzger [25]. It is obvious that consideration of the valency [26,27] situation is critical in any attempt to measure affinity constants or related properties. It has to be stressed that in our work, only monovalent antigens, in particular haptens, were considered. Also, the application of this approach is intended to be applied to monoclonal antibodies without significant affinity heterogeneity. Any use for polyclonal antibodies has to be interpreted with care [28], although we found the application useful [29], except that the resulting value is not an affinity constant in the strict sense. Since haptens possess only one epitope per se, a multivalent situation can be excluded in nearly all cases. A very good discussion of the difference between haptens (such as DNP–lysine) and multivalent antigens has been published by Hornick and Karush [16]. Futhermore, it has to be noted that we do not measure the affinity between the conjugate and the (bivalent) antibody, but the inhibition with a monovalent competitor in a competitive immunoassay.

A very traditional method for affinity determination is the use of the quenching of the intrinsic tryptophan fluorescence of proteins [30,31]. However, this method also seems to be not trivial to apply and hence many of the results might be not reliable [32].

Another well-established method is known as equilibrium dialysis [31]. It was introduced by Marrack and Smith in 1932 [33]. Commercial dialysis chambers of different formats are available. Also, a 3D printed device was published [34]. This method is useful for the binding analysis of small molecules and ions to proteins and other substances of high molecular mass.

Recently, a new technique termed microscale thermophoresis (MST) [35] marketed by Nanotemper seems to have gained some popularity [36]. MST is a homogeneous method and does not need any immobilized reagents. Hence, surface or diffusion issues are much less relevant here. For MST, however, a reagent usually has to be fluorescently labeled, and kinetic data cannot be obtained directly in a similar way to SPR. However, the method is a good addition to the portfolio of methods, which can be used for the technically complementary determination of affinity constants.

Furthermore, many other different methods have been used for this purpose, such as nuclear magnetic resonance (NMR) [37], fluorescence anisotropy, also known as fluorescence polarization [38,39], and many others [40,41,42,43]. Also, quite popular is the use of immunoassays of different formats for the “estimation” of affinity constants or the determination of relative affinities. However, in many cases, it remains unclear how different these results are from the thermodynamic equilibrium constant, and often only relative values are given. The most well-known method seems to be the approach of Friguet [44]. It can be considered as label-free, and a solid phase is only used to trap some of the antibodies. Although this method is occasionally used, some limitations seem to prevent its widespread use [45]. A method which is similar to our approach was proposed by Beatty et al. [46]. In this case, the direct interaction between an immobilized antigen and an antibody is determined with the help of a labeled secondary antibody. Although this simple and fast approach is attractive, the method seems to neglect the complex influence of multivalency caused by adsorbed antigen [47], which is most likely relevant in this non-competitive format. Moreover, it depends on several assumptions that usually cannot be confirmed.

In our paper, we propose a simple method to use immunoassays of different formats to determine affinity constants, which seems to be able to obtain a value, which is quite close to the numbers obtained with other, more complex methods. This approach is based on the idea that the IC_50_ values (fitted midpoint of the 4-parameter equation) of competitive assays converge towards the reciprocal affinity constant when the reagents are diluted to infinity [48,49]. Of course, this is not possible in practice; however, the convergence trend shows how closely the experimental value approached the affinity constant. This convergence is tested by further dilution of the reagents. In most cases, two reagents are relevant in this context. Hence, a 2-dimensional dilution array is necessary. In the best case, you only need four (or nine) calibration curves for a final result. If no convergence is reached yet, the experiment can be repeated with lower concentrations of the reagents until convergence is achieved. There are several advantages of this approach: The experiment is very simple and fast; different formats can be used; only one (absolute) concentration needs to be known, the molar concentration of the analyte; and transparent evaluation of the data. Due to the competitive mechanism with monovalent analytes, issues with multivalency were not observed. Many other methods need complicated calculations and corrections, which might lead to unintended errors, or the user has to trust blindly in the values generated by commercial software.

## 2. Experimental Design

**Figure 1:**
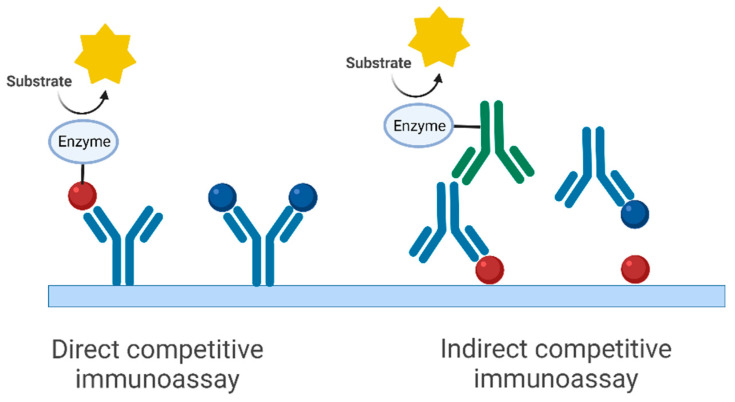
Immunoassay formats used for the approach presented (simplified schemes). The corresponding affinity constant refers to the complex in blue (antibody and antigen/hapten). We do not assume any cooperativity between the two binding sites of the antibody, due to the large distance. The red spheres symbolize haptens that are conjugated with an enzyme label (horseradish peroxidase) or a carrier protein (here BSA), also known as coating antigen. The (different) affinity between the red spheres and the antibody does not play an essential role for the calculated affinity constant. The green antibody is a peroxidase-labeled secondary anti-mouse antibody. Although we did not observe any effects of potential multivalence or cooperativity, multivalent binding of the antibody is avoided anyway due to the very low concentration of the coating antigen in the relevant measurements. Horseradish peroxidase, which was also used as a marker in the direct competitive immunoassay, generally has very low conjugation ratios of about 1, which also greatly reduces the potential effect of multivalence (see “Synthesis of FLAG conjugates”). (Created with BioRender.com).

In this paper, a model system was used to test different ways to determine affinity constants. For this purpose, the monoclonal antibody against the target FLAG, a peptide-based label [50], was chosen. This mouse antibody (IgG_1_) excreted from the clone M2 [51,52,53,54,55,56] is commercially available and is used quite frequently. However, not too many efforts have been made to determine its affinity to its antigen, FLAG. In this work, two different immunoassay formats have been tested (Figure 1), a direct competitive immunoassay (antibody-immobilized, enzyme-labeled FLAG) and an indirect competitive immunoassay (FLAG-immobilized, antibody unlabeled, secondary antibody enzyme-labeled). Since these assays are competitive ones, they are preferentially used for small molecules (haptens). In our case, the target (and competitor) was the 8-amino acid peptide FLAG with the sequence Asp-Tyr-Lys-Asp-Asp-Asp-Asp-Lys (DYKDDDDK).

A typical procedure would be as follows:

Nine IC_50_ values need to be determined on three ELISA 96-well microplates. Each measurement would be obtained in quadruplicate, which seems to be a good compromise between effort and reliability. A calibration curve consisting of eight different concentrations of the analyte would be prepared (usually including the blank value). In most cases, dilution steps of a factor of ten would be adequate to cover a large concentration range. Otherwise, preliminary experiments to determine the optimal concentration steps would be required. In many cases, the development of an assay already delivered useful starting concentrations for the calibration curve. However, some steps in the lower concentration range should be planned, because the sigmoidal curve will shift to lower concentrations during this process. In Table 1, a typical plate setup is shown. Three of these plates would be required to vary the second reagent, too.

**Figure 2:**
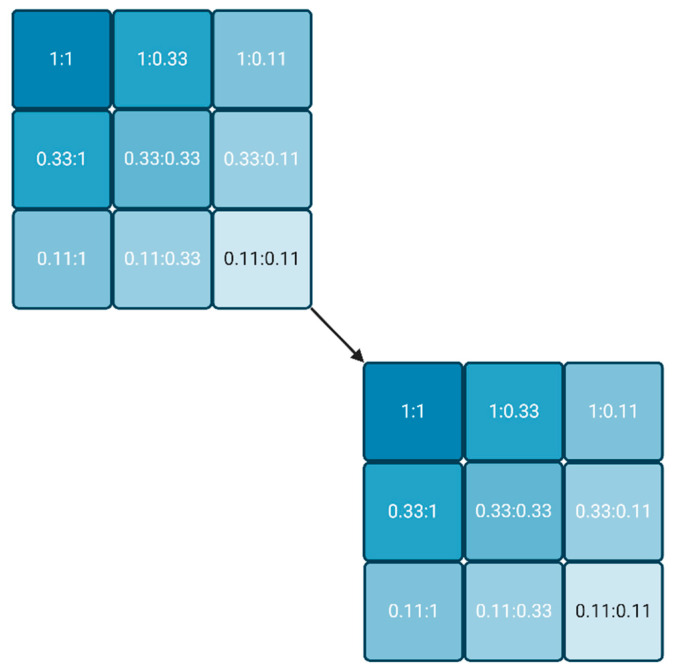
Dilution scheme for two reagents, starting from any two concentrations (1:1) in steps of factor 3. If no convergence of the molar IC_50_ is achieved in the last experiment (0.11:0.11), the process may be repeated with a new cycle (cascade), whereby the relative concentration 0.11 is set to 1. (Created with BioRender.com).

The lowest reagent concentrations are the most interesting, as long as a significant signal can be measured. If a converging IC_50_ is obtained, the lowest IC_50_ already delivers the K_d_ value, or at least a good estimate. If the IC_50_ values do not achieve convergence, the whole approach can be repeated (cascaded) with another series of three plates, usually starting with the lowest reagent combinations of the first round (Figure 2). The process stops when either a convergent IC_50_ is obtained, or the signal is not detectable anymore. The latter condition underlines that the most sensitive labels are preferable for this approach.

Protocol for the direct enzyme immunoassay (antibody-immobilized):

High-binding, flat-bottom microtitration plates (MTP, Greiner) are first coated with donkey anti-mouse antibodies diluted 1:1000 in PBS overnight (100 µL per well). After washing the plate with PBS–Tween 20 three times (300 µL per well), the surface is blocked with 1% of BSA in PBS for 1 h (200 µL per well). After a repeating washing step, different dilutions of the primary antibody M2 are added in PBS/BSA/Tween 20 (100 µL per well, pH 7.45, 0.1% BSA, 0.01% Tween 20) and incubated for 1 h under shaking. After a subsequent washing step, 100 µL per well of FLAG peptide solutions (calibration curve) in PBS/BSA/Tween 20, directly followed by 100 µL per well of FLAG-HRP conjugate were incubated for one hour. After the next washing step, 100 µL of TMB substrate (Seramun, Heidesee, Germany) is added and incubated for up to 45 min. Then, 100 µL of 0.25 M H_2_SO_4_ is added to stop the reaction. The plate is measured at 450 nm in an MTP reader. All buffers are filtered through a syringe filter (0.45 µm) or equivalent.

Protocol for the indirect enzyme immunoassay (antigen-immobilized):

High-binding, flat-bottom microtitration plates (MTP, Greiner, Kremsmünster, Austria) are first coated with FLAG-BSA conjugate diluted 1:1500, 1:4500, and 1:13,500 in PBS overnight (100 µL per well). After washing the plate with PBS–Tween 20 three times (300 µL per well) different dilutions of the FLAG peptide in PBS/BSA/Tween 20 (100 µL per well, pH 7.45, 0.1% BSA, 0.01% Tween 20) and subsequently the primary antibody M2 diluted in PBS/BSA/Tween 20 (100 µL), are added and incubated for 1 h under shaking. After the next washing step, 100 µL of anti-mouse-HRP (1:40,000 in PBS/BSA/Tween 20) is incubated for 1 h, followed by the next washing step. A 100 µL measure of TMB substrate (Seramun) is added and incubated for up to 45 min. Then, 100 µL of 0.25 M H_2_SO_4_ is added to stop the reaction. The plate is measured at 450 nm in an MTP reader. All buffers are filtered through a syringe filter (0.45 µm) or equivalent.

Synthesis of FLAG conjugates:

The conjugates were prepared based on Cys-FLAG (H-CDYKDDDDK-NH_2_). Maleimide-activated HRP (Thermo, Waltham, MA, USA) was dissolved in PBS to obtain a concentration of 1 mg/mL and mixed with Cys-FLAG in PBS (1 mg/mL) and incubated three hours at room temperature and overnight at 4 °C. The conjugate was purified on a PD Spin Trap GD-25 column. MALDI-MS measurements showed a conjugation ratio of about 1:1 for the FLAG-HRP product.

The BSA conjugate was prepared in PBS adjusted to pH 8. First, a BSA solution of about 1 mg/mL was prepared. Then, SBAP (succinimidyl 3-(bromoacetamido)propionate) was dissolved in DMSO to obtain a solution of 1 mg/mL. A 150 µL measure of the BSA solution was slowly mixed with 15 µL of the SBAP solution and incubated at 4 °C for one hour. The conjugate was purified on a PD Spin Trap GD-25 column. Subsequently, a 1 mg/mL concentration of Cys-FLAG was prepared in purified water. A 150 µL measure of the BSA-SBAB conjugate was mixed with 55 µL of the Cys-FLAG solution and incubated for two hours at room temperature and overnight at 4° C. Then, the BSA-SBAP-Cys-FLAG conjugate was purified on a PD Spin Trap GD-25 column equilibrated with PBS (pH 7.4). MALDI-MS measurements showed a conjugation ratio of about 3:1 for the FLAG-BSA product.

## 3. Results and Discussion

The test midpoints (IC_50_) of two different formats have been determined according to the approach described above.

For direct (antibody-immobilized) assays, the concentration of antibody and the concentration of conjugate (hapten-label) were diluted in three steps, respectively, which leads to nine different reagent combinations. Please note that only relative concentrations of the reagents are needed. We suggest a dilution of a factor of 3, which is large enough to be significant, but small enough not end up in extremely low signals. The starting concentrations of the antibody/conjugate pair is somewhat arbitrary and should be chosen according to previous experience with the respective assay. An optimized ELISA might be obtained with the help of a checkerboard titration, if performed anyway. As an orientation, in our assays the concentration of the antibodies and of the conjugate were 11 ng/mL, respectively. If a biointeraction is used for the oriented immobilization of the antibodies or other reagents (e.g., streptavidin/biotinylated antibody, protein A or G, anti-IgG), these reagents are not subject to this approach and can be chosen more or less arbitrarily, since they are not directly involved in the competitive step.

For indirect (antigen-immobilized) assays, the concentration of antigen (or hapten) and the concentration of antibody were also diluted in three steps, respectively, which also leads to nine different reagent combinations. Similar to the direct immunoassays, any secondary reagents, such as enzyme-labeled antibodies, are not subject of the dilution steps, only reagents directly involved in the competition are relevant here. As an orientation: in our assays, the starting concentration of the hapten–BSA (conjugation density: ≈3.5) conjugate was 0.044 ng/mL and 33.3 ng/mL for the antibody.

The concentration of the FLAG peptide was determined gravimetrically, whereby no impurities from water, salts, or counterions such as trifluoroacetates were taken into account. Since the concentration of the antigen is crucial for the accuracy of the affinity constant, as it is directly reflected in deviations from the “true” affinity constant, the impurities should be taken into account if a high accuracy of the result is important. The most common method for a reliable determination of the peptide concentration seems to be amino acid analysis, e.g., [57,58,59,60], which is sometimes offered by companies performing custom peptide synthesis. If an identical calibration substance of known purity is available, many other methods might be suitable [61], such as HPLC with UV detection.

In Table 2, molar IC_50_ values of nine calibration curves of the direct, competitive immunoassays are shown. Usually, the IC_50_ decreases with the dilution of the reagents. However, by approaching the strictly affinity-controlled regime, the IC_50_ converges and finally remains constant. The final IC_50_ is the basis for the calculation of K_d_. There is a small issue with this method. In both assay formats, the analyte is diluted with the reagent by a known factor, based on the volumes of the reagents. Due to pre-incubation effects (“cold start”) and different diffusion coefficients of the analyte and the reagent, their kinetic behavior might be different. Usually, it is not known whether the equilibrium is achieved or not. Therefore, there is a small uncertainty about to which extent the dilution of the analyte has to be corrected. To minimize this effect, a relatively large volume of the analyte (e.g., 200 µL) and a small volume of the reagent (e.g., 20 µL) might be used. Furthermore, relatively long incubation times should be used for the competitive step (at least 1 h). In this case, a correction factor for the IC_50_ of 0.91 would be adequate. The effect of incubation time was previously discussed [62]. However, considering the huge discrepancies of reported affinity constants in different papers, this small difference seems to be of minor relevance. It also has to be considered that affinity constants are dependent on the environment of the complex and particularly on the temperature. Therefore, only affinity constants determined under standardized conditions can be expected to be identical.

In Table 3, the nine calibration curves (shown in the Supplementary Materials) of the indirect, competitive immunoassays also delivered nine molar IC_50_ values. Perhaps surprisingly, the converged IC_50_ values of both methods are essentially the same (highlighted with grey background), although the assay format is quite different. This is a strong indication that these IC_50_ values really represent an affinity constant, which is a characteristic of the respective system. We do not claim that these values are identical to a strict thermodynamic derivation but are “fit for purpose” in research and commercial quality control. We are confident that these values are practically useful and facilitate to compare antibodies and their properties, not only in a relative way. In addition, this approach is so fast and simple that essentially all antibodies could be characterized this way without excessive investments and workload.

Table 4 compares the results of our work with the available data from the literature. These figures appear to be sufficiently consistent in view of the large deviations found in the literature. These results lead to some interesting conclusions. Due to the good agreement of the two formats and the values from the literature, some objections can be dismissed. Influence of multivalency is very unlikely due to the use of a monovalent analyte and the fact that otherwise the values would diverge typically by a factor of 100 or even more. The direct immunoassay format with a hapten–peroxidase conjugate shows monovalent behavior, due to the very low conjugation levels due to the low number of accessible lysines (1–2 amino groups). Hapten conjugates based on other proteins, such as BSA, also seem to show a low tendency for multivalent complexes, since even multivalent conjugates may allow only monovalent binding on a surface due to size and steric considerations. Low coating concentrations or preferably blending with non-conjugated carrier will reduce the risk of multivalent binding even more. Since the proposed method relies on the maximally diluted reagents, this situation automatically minimizes any situation of crowded antigens or conjugates. Surprisingly, neither the conjugation density nor the conjugate and antibody concentration need to be known. Only relative dilutions are used for this approach. The only absolute concentration needed is that of the analyte.

According to model calculations [48], the IC_50_ drops proportionally to the dilution of the respective reagent, as long as the assay is governed by the concentration regime. This means that in this case, large dilution steps are required to reach the signal-limited range. In this regime, the IC_50_ converges to a constant value [65]; however, the signal drops proportionally to the dilution, until the limit of detection of the label is reached.

If both dilution steps of reagent A and B, and their combination, lead to the same IC_50_, the affinity limited range is reached, and the affinity constant has been determined. In this work, it should be examined, whether this approach leads to the same affinity constant, irrespective of the starting point and the immunoassay format, which was indeed the case. To avoid misunderstandings: in any practical application, only one format is required to determine the affinity constant. Two formats were used here for validation purposes only.

In some cases, particularly with antibodies of extremely high affinity, the IC_50_ values might not converge sufficiently, when the signal has reached the limit of detection. In these cases, the lowest IC_50_ obtained in these experiments determines the minimum affinity constant (maximal K_d_). This situation is not uncommon; most methods used for the determination of affinities show a limit to high affinities (or very low K_d_) values. This is caused by the fact that the relevant analyte concentrations become lower and lower and hence cannot be quantified precisely anymore. This also means that more powerful (sensitive and accurate) methods for the determination of the respective conjugates will lead to an improvement for this approach, too. A final word of encouragement: very low signal intensities do not mean that the measurement is useless or even has failed. Experimental reproducibility, as measured by the variability of replicates, is often surprisingly good, even with very low signals. However, some limitations should be noted. The proposed method should not be used to determine affinity constants of oligomeric antigens, antigens with homologous domains, or multivalent hapten conjugates. Furthermore, non-competitive test formats such as sandwich immunoassays, or direct immunoassays such as Western blots, are generally not suitable for this approach.

## Figures and Tables

**Table 1:** Hypothetical setup of typical ELISA calibration curves on a 96-well microtitration plate (MTP) in order to determine three IC_50_ values. At least three of these plates are required in which the second reagent is also varied from 1 to 1:3 and finally 1:9.

	Variation of Reagent Concentration (Either Antibody or Conjugate), Second Reagent Is Kept Constant		
	1 (Start)	1:3	1:9
**Analyte** concentration (mol/L)	10-3	10-3	10-3
	10-4	10-4	10-4
	10-5	10-5	10-5
	10-6	10-6	10-6
	10-7	10-7	10-7
	10-8	10-8	10-8
	10-9	10-9	10-9
	10-10	10-10	10-10
	10-11	10-11	10-11
	10-12	10-12	10-12

**Table 2:** IC_50_ values in nM by direct, competitive ELISA.

Direct, Competitive ELISA Competitor: FLAG Peptide		Conjugate HRP-FLAG (Tracer)		
		1:90,000 (Start)	1:270,000 (1:3)	1:810,000 (1:9)
**Antibody M2**	**1:90,000 (start)**	166 ± 21 nM	139 ± 28 nM	121 ± 30 nM
	**1:270,000 (1:3)**	147 ± 23 nM	133 ± 42 nM	90 ± 13 nM
	**1:810,000 (1:9)**	127 ± 18 nM	126 ± 73 nM	90 ± 37 nM

**Table 3:** IC_50_ values in nM by indirect, competitive ELISA.

Indirect, Competitive ELISA Competitor: FLAG Peptide		Conjugate BSA-FLAG (Immobilized)		
		1:1,500 (Start)	1:4,500 (1:3)	1:15,000 (1:10)
**Antibody M2**	**1:30,000 (start)**	169 ± 21 nM	146 ± 10 nM	160 ± 16 nM
	**1:90,000 (1:3)**	121 ± 16 nM	104 ± 8 nM	121 ± 16 nM
	**1:270,000 (1:9)**	109 ± 12 nM	93 ± 5 nM	93 ± 15 nM

**Table 4:** Affinity constants of the antibody M2 to the FLAG peptide determined by different methods.

Method	K_d_ (nM)	FLAG Sequence	Conjugate	Reference
direct IA	90 ± 37	DYKDDDDK	HRP-CDYKDDDDK	This work
indirect IA	93 ± 15	DYKDDDDK	BSA-CDYKDDDDK	This work
FPIA	150	-	5-FAM-SGSGDYKDDDDK	[63]
SPR	50 ± 30	DYKDDDDK	M2 (immobilized)	[64]

## Data Availability

Some data are given in the Supplementary Materials.

## Supplementary Materials

The following supporting information can be downloaded at: https://www.mdpi.com/article/10.3390/mps7030049/s1, Nine calibration curves of indirect competitive immunoassays.
