# Supplementary material for: Simple Determination of Affinity Constants of Antibodies by Competitive Immunoassays
Source: Methods Protoc. 2024 Jun 13;7(3):49. doi: 10.3390/mps7030049 (PMC11206456; doi:10.3390/mps7030049)

Supplement

Simple determination of affinity constants of antibodies by competitive immunoassays

Janina Fischer [1,2], Jan Ole Kaufmann [1,3], and Michael G. Weller [1,2,*]

[1] Federal Institute for Materials Research and Testing (BAM), Richard-Willstätter-Strasse 11, 12489 Berlin, Germany
[2] Department of Chemistry, Humboldt-Universität zu Berlin, Brook-Taylor-Straße 2, 12489 Berlin, Germany
[3] Charité – Universitätsmedizin Berlin, Charitéplatz 1, 10117 Berlin, Germany
* Correspondence: michael.weller@bam.de; Tel.: +49-30-8104-1150

Nine calibration curves of indirect competitive immunoassays

Antigen: FLAG peptide; Antibody: Clone M2; Conjugate: BSA-FLAG

MTP1, 0.044 µg/ml BSA-FLAG

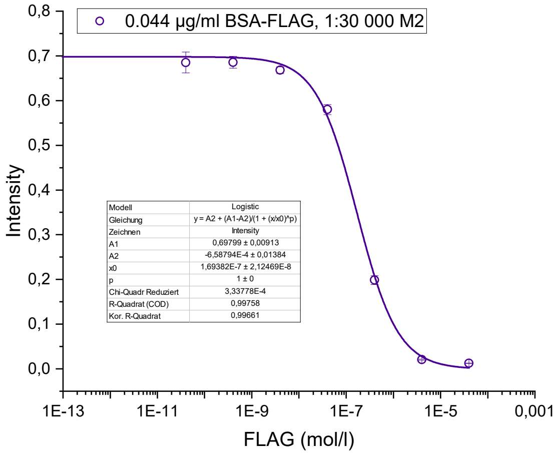

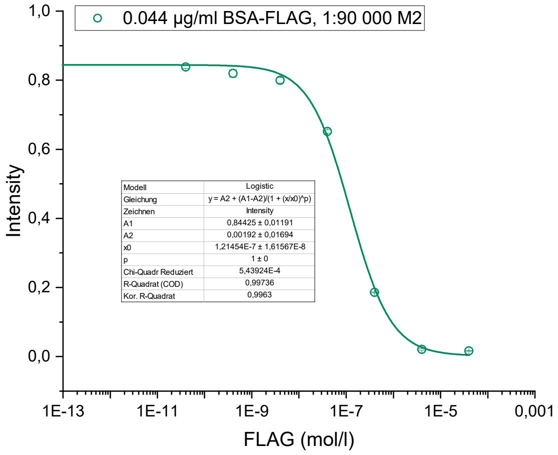

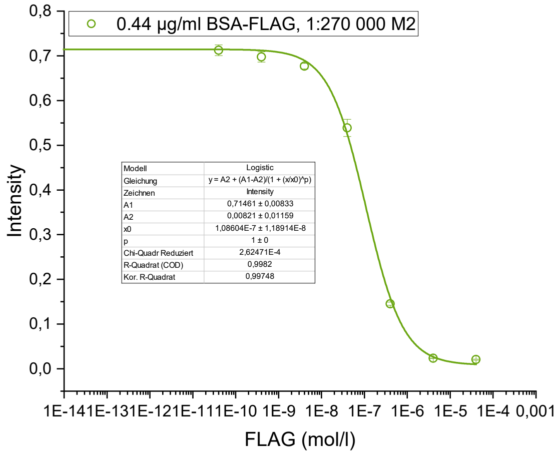

MTP2, 0.0133 µg/ml BSA-FLAG

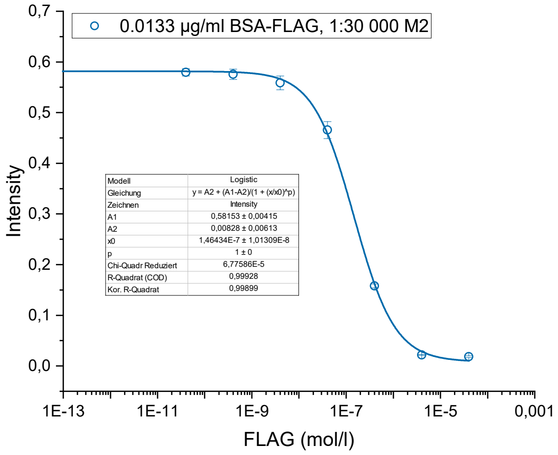

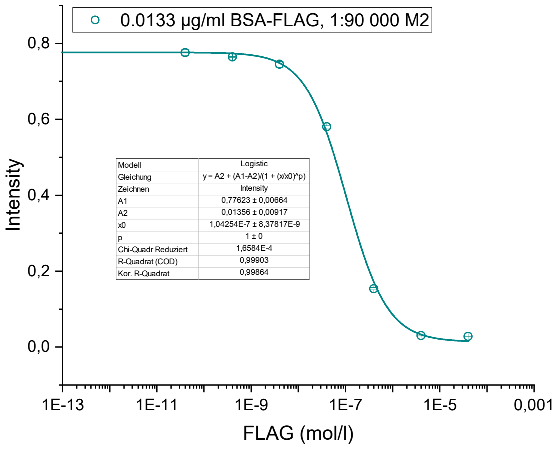

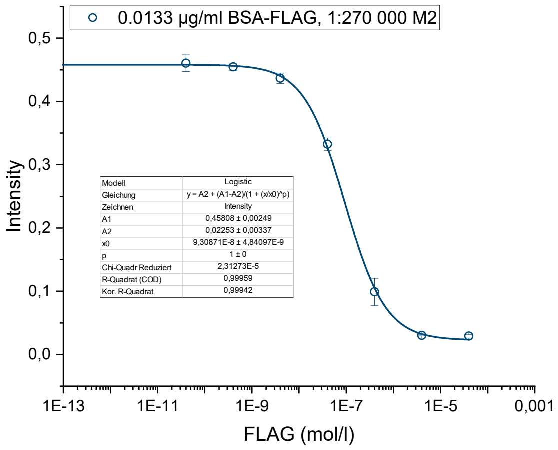

MTP3, 0.0044 µg/ml BSA-FLAG

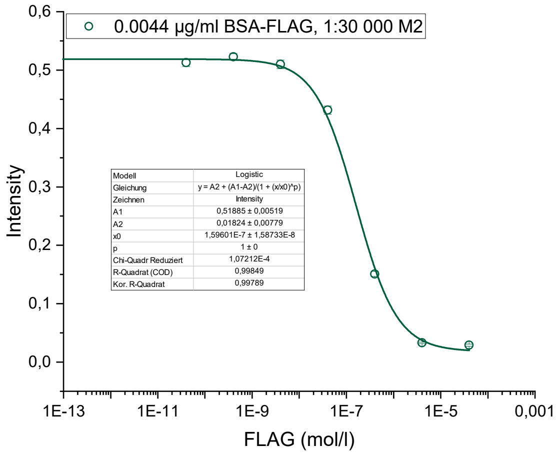

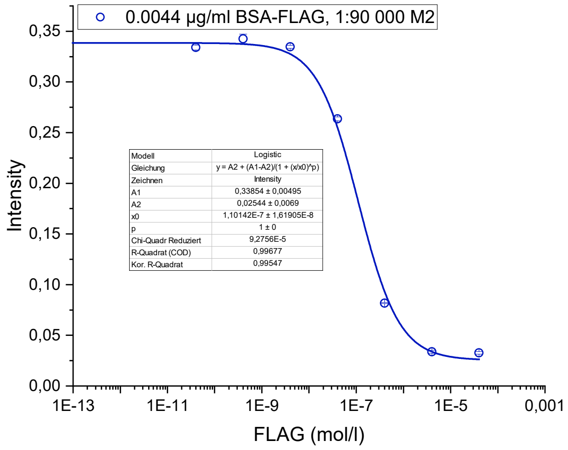

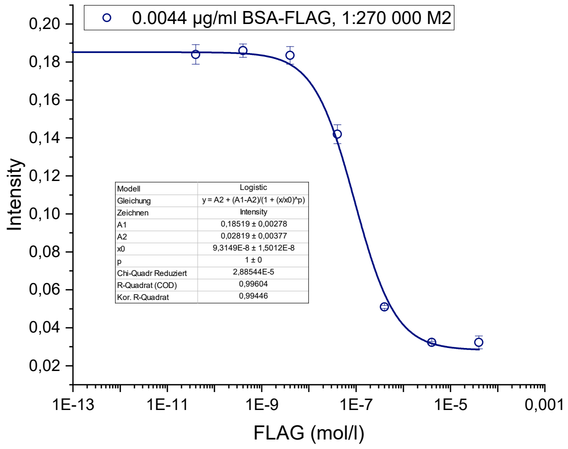

Supplement: Supplementary file 1 [file mps-07-00049-s001.zip › mps-2989473-supplementary.pdf]
